# Sex-Specific Response of the Brain Free Oxylipin Profile to Soluble Epoxide Hydrolase Inhibition

**DOI:** 10.3390/nu15051214

**Published:** 2023-02-28

**Authors:** Jennifer E. Norman, Saivageethi Nuthikattu, Dragan Milenkovic, John C. Rutledge, Amparo C. Villablanca

**Affiliations:** 1 Division of Cardiovascular Medicine, Department of Internal Medicine, University of California, Davis, CA 95616, USA; 2 Department of Nutrition, University of California, Davis, CA 95616, USA

**Keywords:** oxylipin, soluble epoxide hydrolase, brain, cognitive function, dementia, sex differences

## Abstract

Oxylipins are the oxidation products of polyunsaturated fatty acids and have been implicated in neurodegenerative disorders, including dementia. Soluble epoxide hydrolase (sEH) converts epoxy-fatty acids to their corresponding diols, is found in the brain, and its inhibition is a treatment target for dementia. In this study, male and female C57Bl/6J mice were treated with an sEH inhibitor (sEHI), trans-4-[4-(3-adamantan-1-yl-ureido)-cyclohexyloxy]-benzoic acid (t-AUCB), for 12 weeks to comprehensively study the effect of sEH inhibition on the brain oxylipin profile, and modulation by sex. Ultra-high-performance liquid chromatography–tandem mass spectrometry was used to measure the profile of 53 free oxylipins in the brain. More oxylipins were modified by the inhibitor in males than in females (19 versus 3, respectively) and favored a more neuroprotective profile. Most were downstream of lipoxygenase and cytochrome p450 in males, and cyclooxygenase and lipoxygenase in females. The inhibitor-associated oxylipin changes were unrelated to serum insulin, glucose, cholesterol, or female estrous cycle. The inhibitor affected behavior and cognitive function as measured by open field and Y-maze tests in males, but not females. These findings are novel and important to our understanding of sexual dimorphism in the brain’s response to sEHI and may help inform sex-specific treatment targets.

## 1. Introduction

Oxylipins are products of polyunsaturated fatty acids (PUFAs), produced through oxidation via cytochrome p450 (CYP), lipoxygenase (LOX), and cyclooxygenase (COX) or non-enzymatic oxidation pathways [1]. Studies have found that oxylipins have a role in many biological processes and diseases, including inflammation, metabolic diseases, and cardiovascular diseases [2,3,4,5,6,7]. Further, evidence supports a role for oxylipins in modulation of neuroinflammation and neurodegenerative diseases, including dementias [8,9,10,11,12].

Soluble epoxide hydrolase (sEH), is involved in the metabolism of certain oxylipins. Specifically, sEH converts epoxy-fatty acids produced by CYP enzymes into their corresponding diols, those produced from arachidonic acid are epoxyeicosatrienoic acids (EpETrEs) and their corresponding diols, dihydroxyeicosatrienoic acids (DiHETrEs) [13]. sEH is found in many tissues, including the brain [14,15]. Within the brain, sEH is found in many regions and within various cell types, including glial cells, neurons, and vascular cells [15]. Research on sEH has implicated it as a treatment target for many disease types, including metabolic, inflammatory, and cardiovascular diseases [13,16,17,18,19,20]. Further, sEH has been associated with, and the inhibition of its activity has been proposed as a treatment target for, diseases of the brain, including dementias [9,21,22,23,24,25,26,27,28,29,30,31,32,33,34,35,36,37,38,39,40]. sEH inhibition has been demonstrated to positively impact modulation of neuronal activity, glial cell activity, cell survival, neuroinflammation, blood flow, and blood–brain barrier permeability; further, it has been suggested that many of these benefits are due to the subsequent increase in epoxy-fatty acid levels, in particular, the levels of EpETrEs [41,42,43,44,45,46,47]. 

Studies have shown sex differences in sEH expression and activity in multiple tissues, including the brain [48,49,50]. In rodents, females have been shown to have lower expression of sEH in the whole brain and cerebral vessels compared to males, as well as smaller infarct volumes after middle cerebral artery occlusion (MCAO) [49,50]. In line with this, others have found that transgenic mice, expressing human sEH in endothelial cells, exhibited impaired endothelial-dependent vasodilation, and that the effect of induced expression of sEH was more pronounced in females [47]. These findings have been supported by in vitro work as well. In endothelial and neuronal cultures, cells derived from males exhibited higher levels of sEH and were more susceptible to ischemic damage than cells derived from females [51,52]. Evidence indicates that these sex differences in sEH and their downstream consequences are due, at least in part, to sex hormones. It has been demonstrated that 17β-estradiol suppresses cerebral sEH expression and that ovariectomy abolishes the sex differences in infarct size after MCAO [50,53].

Given the sex differences in levels of the sEH enzyme, it would be likely that the response to deficiency or inhibition of sEH would be sex-dependent, and this is supported by the literature. Genetic deletion of sEH reduces infarct volumes in males after MCAO, but has no effect in females [49,50]. Further, we have previously demonstrated a sex-dependent response of the microvascular transcriptome to sEH inhibition [54]. Thus, studies investigating the therapeutic potential of targeting sEH need to address males and females separately and not generalize results from one sex to the other or not attend to biologic sex.

Although the substrates and products of sEH are often examined, to our knowledge, the effect of inhibiting sEH on the overall brain free oxylipin composition has not been investigated. In this study, we therefore sought to characterize the effect of inhibiting sEH on the free oxylipin profile of the brain. We chose to examine the brain free oxylipin pool, as these are generally seen as the biologically active form [1]. Further, given the substantial evidence for sex differences in sEH expression and response to its modulation, we examined the impact of sEH on the brain free oxylipin profile in each sex separately. We hypothesized that providing mice with an sEH inhibitor (sEHI) would alter the brain oxylipin profile towards a neuroprotective, anti-inflammatory profile, which would be more pronounced in males.

## 2. Materials and Methods

**Figure 1:**
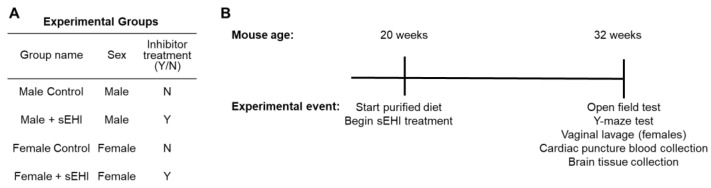
Experimental groups and study timeline: (**A**) Table of experimental groups detailing sex and inhibitor treatment. (**B**) Timeline of experimental procedures in relation to mouse age.

An overview of the experimental groups and the experimental timeline can be found in Figure 1. The experimental details follow in the sections below.

### 2.1. Animals

Research was conducted in conformity with the Public Health Service Policy on Humane Care and Use of Laboratory Animals and ARRIVE 2.0 guidelines [55] and was approved by the Institutional Animal Care and Use Committee of the University of California, Davis (protocol number 20943, approval date 18 April 2019). Mice were housed in a temperature- and humidity-controlled environment with a 12 h light/dark cycle at the University of California, Davis Mouse Biology Program. Female mice were housed with up to three mice per cage; male mice were housed singly. Activity, water, and food intake were monitored by vivarium staff to ensure the well-being of the mice.

Male and female C57Bl/6J mice were purchased from Jackson Laboratories (stock 000664). From 20 weeks of age, all mice were fed a commercially available purified diet (catalog number TD.08485 from Envigo Teklad Diets, Madison, WI, USA), provided *ad libitum*. The macronutrient content of the diet was as follows: 13% kcal from fat, 19.1% kcal from protein, and 67.9% kcal from carbohydrates.

### 2.2. Soluble Epoxide Hydrolase Inhibitor (sEHI)

One group of male mice (n = 7) and one group of female mice (n = 7) were treated with the soluble epoxide hydrolase inhibitor (sEHI), trans-4-[4-(3-adamantan-1-yl-ureido)-cyclohexyloxy]-benzoic acid (t-AUCB, Cayman Chemical, Ann Arbor, MI, USA). The t-AUCB was provided in the drinking water using polyethylene glycol 400 (PEG400) (Millipore, Burlington, MA, USA) as a vehicle from 20 weeks of age until sacrifice at 32 weeks of age (Figure 1B). We chose this timeline of treatment and sacrifice to align with our previous publications regarding sEHI, which found this length of treatment sufficient to induce changes in the brain microvascular transcriptome [54,56,57]. Consistent with prior protocols, the final contents of the drinking water were 1% (by volume) PEG400 and 10 mg/L t-AUCB [58,59]. In agreement with others, mice consumed approximately 7 to 7.5 mL of water each day [60]. This resulted in mice consuming approximately 2.5 to 3 mg of t-AUCB per kg per day. For control groups, one group of male mice (n = 7) and one group of female mice (n = 7) were not treated with t-AUCB. The PEG400 vehicle was not added to drinking water of these control mice; however, 1% PEG400 is a low amount and in prior studies has been shown to not have a biological effect [61]. Overall, there were four experimental groups of mice: (1) male control (those not receiving sEHI), (2) male + sEHI (treated with sEHI), (3) female control (those not receiving sEHI), and (4) female + sEHI (treated with sEHI) (Figure 1A).

### 2.3. Assessment of Estrus Cycle Phase

For all female mice, vaginal lavage with phosphate-buffered saline (PBS) was performed at the end of the study after administration of anesthesia and prior to sacrifice. The PBS with collected vaginal cells was then applied to a glass slide and allowed to dry. Slides were stored at room temperature until staining with 0.1% crystal violet. Phase of estrus cycle was assessed by examining the stained cells using light microscopy. Samples were categorized as proestrus, estrus, metestrus, or diestrus based on the cell types observed as previously described [62].

### 2.4. Tissue Collection

At the end of the study period, mice were fasted for 8 h before being anesthetized with a combination of Ketamine and Xylazine. Blood was collected by ventricular puncture under anesthesia, then mice were euthanized. The brain was immediately harvested and snap-frozen in liquid nitrogen to preserve the lipid profiles and integrity. We estimate the total time for this process was less than 5 min. Samples remained in frozen storage (at −80 °C) until extraction of the oxylipins.

### 2.5. Serum Analyses

Serum was separated from whole blood by centrifugation and samples were stored at −80 °C until analysis. Glucose and total cholesterol were measured using enzymatic assays from Fisher Diagnostics (Middleton, VA, USA). Insulin was determined by electrochemiluminescence from Meso Scale Discovery (Rockville, MD, USA). All assays were performed in triplicate by the University of California Davis Mouse Metabolic Phenotyping Center (UCD MMPC).

### 2.6. Mouse Behavioral and Cognition Testing

We assessed behavior and cognitive function utilizing the open field test and the Y-maze test. The open field test assesses locomotor activity and anxiety-like behavior in mice, with increased time in the center of the field being indicative of lower levels of anxiety [63]. The Y-maze is widely used to assess spatial and learning memory and assesses the mouse’s active retrograde working memory, by observing how often mice explore the three arms of the maze in succession [64]. 

#### 2.6.1. Open Field Test

Mice were adapted to the testing room for 30 min, then placed in the center of a Columbus Instruments Opto-Varimex 4 for the extent of a 20-min trial. Movement was measured as x, y, and z bream breaks. The perimeter was defined as the outer 8.4 cm region of the 43.5 cm box, or ~60% of total surface area. The open field tests were performed by the UCD MMPC. 

#### 2.6.2. Y-Maze

Mice were adapted to testing room for 30 min, then placed in the center of the Y-maze and were tracked with an overhead camera for the extent of an 8-min trial. An elevated white plastic Y-maze with three 40 cm arms at 120-degree angles. An alternation score was computed as the number of times the three arms were sequentially entered. The % alternation score is the number of alternations divided by maximum alternation triplets. The Y-maze tests were performed by the UCD MMPC. 

### 2.7. Analysis of the Brain Free Oxylipin Profile

We extracted the free oxylipins from the right hemisphere of brain tissue from each mouse for analysis and quantification by ultra-high-performance liquid chromatography–tandem mass spectrometry (UHPLC-MS/MS). The UHPLC-MS/MS measurements were performed once. All measured oxylipins and internal standards with their abbreviations can be found in Table S1. 

#### 2.7.1. Free Oxylipin Extraction from Brain

Oxylipins were extracted from the right hemisphere of brain tissue as previously described [65]. Briefly, the tissue was homogenized using a bead homogenizer and zirconia beads in methanol with butylated hydroxytoluene and acetic acid, spiked with a mixture of deuterated surrogate standards. Homogenized samples were centrifuged. The supernatant was loaded onto solid phase extraction columns, the columns were rinsed, and the oxylipins were eluted with methanol and ethyl acetate. After drying under nitrogen, the oxylipins were reconstituted in 100 µL methanol. 

#### 2.7.2. UHPLC-MS/MS Analysis of Free Oxylipins

Ten µL of the reconstituted free oxylipin extract was analyzed by UHPLC-MS/MS using an Agilent 1290 Infinity UHPLC system coupled to an Agilent 6460 Triple Quadrupole mass-spectrometer (Agilent Technologies, Santa Clara, CA, USA), equipped with an Agilent ZORBAX Eclipse Plus C18 column (2.1 × 150 mm, 1.8 μm particle size; Agilent Technologies, Santa Clara, CA, USA; Cat #959759-902). The specifics of the UHPLC-MS/MS analyses were described previously [65]. Optimization parameters and parent and product ion monitoring pairs are described in Table S2. We have provided the raw mass spectra for the standard, blank, and a representative sample for 15-HETE in Figure S1. 

#### 2.7.3. Data Analysis

Oxylipins with no discernible peaks, or where 50% of all groups had a result of not detected, were removed from analyses. The oxylipins removed from analyses for these reasons were: Resolvin E1, 17-HDoHE, 17(18)-EpETE, 14(15)-EpETE, 11(12)-EpETE, 8(9)-EpETE, 14,15-DiHETE, 11,12-DiHETE, 8,9-DiHETE, 15-HEPE, 8-HEPE, LTB3, PGE3, 8,9-DiHETrE, 20-HETE, LXA4, LTC4, LTE4, 20-OH-LTB4, 20-COOH-LTB4, PGB2, and 13-oxo-ODE. For oxylipins kept in analyses, any non-detects were replaced by 1/5 of the minimum positive value of that variable to estimate the limit of detection. The peaks for PGD1 and PGE1 were indistinguishable; therefore, they were combined and are referred to as PGD/E1. For many samples, PGD2 signals were above the standard curve; the values were included in the analyses. Some samples were lost due to inadvertent breakage of tubes during homogenization of the tissue, which resulted in analysis of brain free oxylipins of n = 6 samples for the male control and male sEHI groups.

### 2.8. Statistical Analysis

Statistical analyses of EpETrE/DiHETrE ratios, body weight, serum parameters, and cognitive/behavioral function were performed using Prism (GraphPad Software, San Diego, CA, USA). Any outliers were determined by ROUT with Q = 1%. Pairwise analyses between control and sEHI groups were performed using t-test (with Welch’s correction if variances were not equal) or Mann–Whitney test (if not normally distributed). Significance was determined at *p* < 0.05.

Statistical analyses of oxylipins were performed using MetaboAnalyst 5.0 [66,67]. All pairwise comparisons of oxylipins between control and sEHI groups were performed on data without transformation using the non-parametric Wilcoxon rank-sum test, since data were not normally distributed for all oxylipins. We used sparse partial least squares discriminate analysis (sPLS-DA), which utilizes the selection of the most discriminative features to classify samples [68], and a hierarchical clustering heatmap to examine all four groups of mice. sPLS-DA and the hierarchical clustering heatmap were performed using log-transformed data. The heatmap was clustered using Euclidean distance and Ward clustering methods. Boxplots were generated using Prism (GraphPad Software, San Diego, CA, USA). Significance was determined at *p* < 0.05 based on the raw *p*-value, when the FDR *p*-value was below 0.05 this was also indicated. 

Spearman correlation analyses of significant oxylipins with the cognitive/behavioral function outcomes were conducted using Prism (GraphPad Software, San Diego, CA, USA). Significance was determined at *p* < 0.05,

## 3. Results

**Figure 2:**
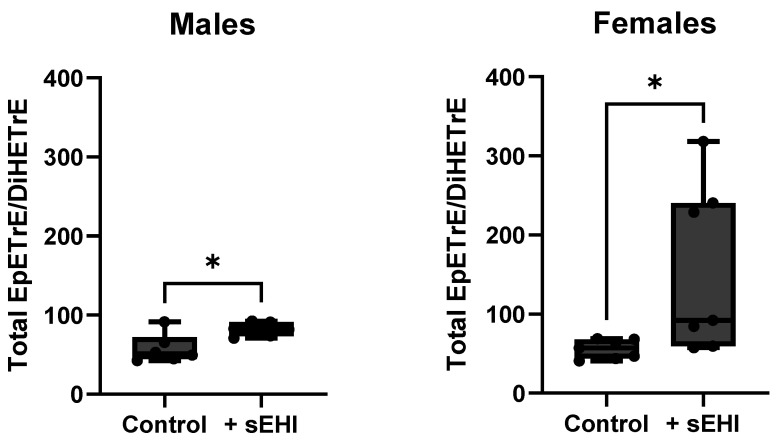
Total EpETrE/DiHETrE ratios of brain free oxylipin contents in males and females. The box extends from the 25th to the 75th percentile, with the middle line indicating the median. The whiskers extend to the minimum and maximum values, with individual values indicated by black dots. For definitions of oxylipin abbreviations see Table S1. * *p* < 0.05.

To confirm that the sEHI dose was sufficient to inhibit sEH, we compared the sEH substrate to product ratio of total EpETrE/DiHETrE of sEHI-treated mice to controls without sEHI for each sex. In both males and females, mice receiving the sEHI had higher EpETrE/DiHETrE ratios than controls (Figure 2).

Body weight did not differ between mice in the sEHI-treated and control groups in both males and females (Table 1). Fasting levels of serum insulin, glucose, and cholesterol did not differ between sEHI-treated and control mice in either sex (Table 1). There were no differences between the sEHI-treated and control females in estrus cycle phase (data not shown).

### 3.1. Effects of sEHI on the Brain Oxylipin Content in Males

**Figure 3:**
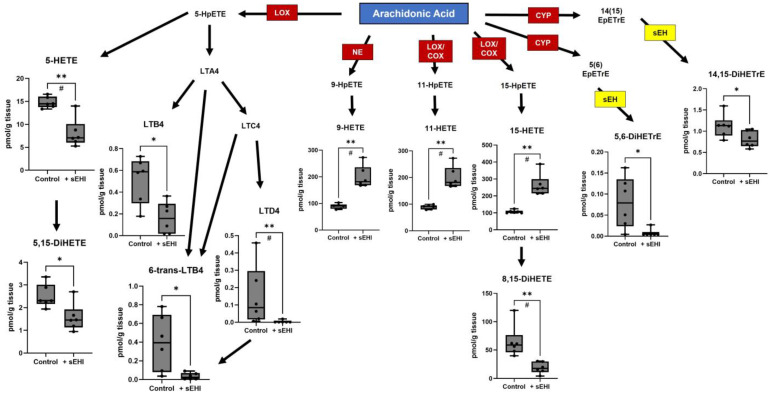
Oxylipins downstream of arachidonic acid altered by sEHI in males. Boxplots of oxylipins that were significantly different in the brains of male sEHI-treated and control mice are shown in a diagram demonstrating the pathways producing the oxylipins from arachidonic acid. The box extends from the 25th to the 75th percentile, with the middle line indicating the median. The whiskers extend to the minimum and maximum values, with individual values indicated by black dots. For definitions of oxylipin abbreviations see Table S1. LOX: lipoxygenase enzyme; NE: non-enzymatic; COX: cyclooxygenase enzyme; CYP: cytochrome p450 enzyme; sEH: soluble epoxide hydrolase; * *p* < 0.05; ** *p* < 0.01; # FDR adjusted *p* < 0.05.

**Figure 4:**
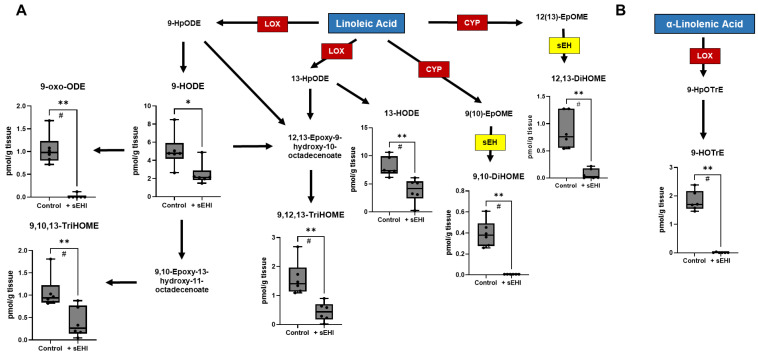
Oxylipins downstream of linoleic acid and α-linolenic acid altered by sEHI in males. Boxplots of oxylipins that were significantly different in the brains of male sEHI-treated and control mice are shown in a diagram demonstrating the pathways producing the oxylipins from linoleic (**A**) and α-linolenic (**B**) acid. The box extends from the 25th to the 75th percentile, with the middle line indicating the median. The whiskers extend to the minimum and maximum values, with individual values indicated by black dots. For definitions of oxylipin abbreviations see Table S1. LOX: lipoxygenase enzyme; CYP: cytochrome p450 enzyme; sEH: soluble epoxide hydrolase; * *p* < 0.05; ** *p* < 0.01; # FDR adjusted *p* < 0.05.

We analyzed the brain free oxylipins of males treated with sEHI as compared to controls (not receiving the inhibitor), using pairwise comparison. There were 19 oxylipins that differed between these two groups in males. Eleven of these oxylipins were products of arachidonic acid metabolism (Figure 3), most of which were downstream of LOX enzymes, including 5-HETE, 5,15-DiHETE, LTB4, 6-trans-LTB4, LTD4, 11-HETE, 15-HETE, and 8,15-DiHETE. Two arachidonic acid products were downstream of CYP enzymes and direct products of sEH (5,6-DiHETrE and 14,15-DiHETrE), and one (9-HETE) was a product of non-enzymatic oxidation. Seven oxylipins were products of linoleic acid metabolism (Figure 4A), most of these were downstream of LOX enzymes, including 9-HODE, 9-oxo-ODE, 13-HODE, 9,10,13-TriHOME, and 9,12,13-TriHOME. Two linoleic acid products were downstream of CYP enzymes and direct products of sEH (9,10-DiHOME and 12,13-DiHOME). One oxylipin was a LOX product of α-linolenic acid metabolism (9-HOTrE) (Figure 4B). Of the 19 oxylipins significantly changed (*p* < 0.05) in sEHI-treated males, the majority were decreased compared to controls, except three (9-HETE, 11-HETE, and 15-HETE).

### 3.2. Effects of sEHI on the Brain Oxylipin Content in Females

**Figure 5:**
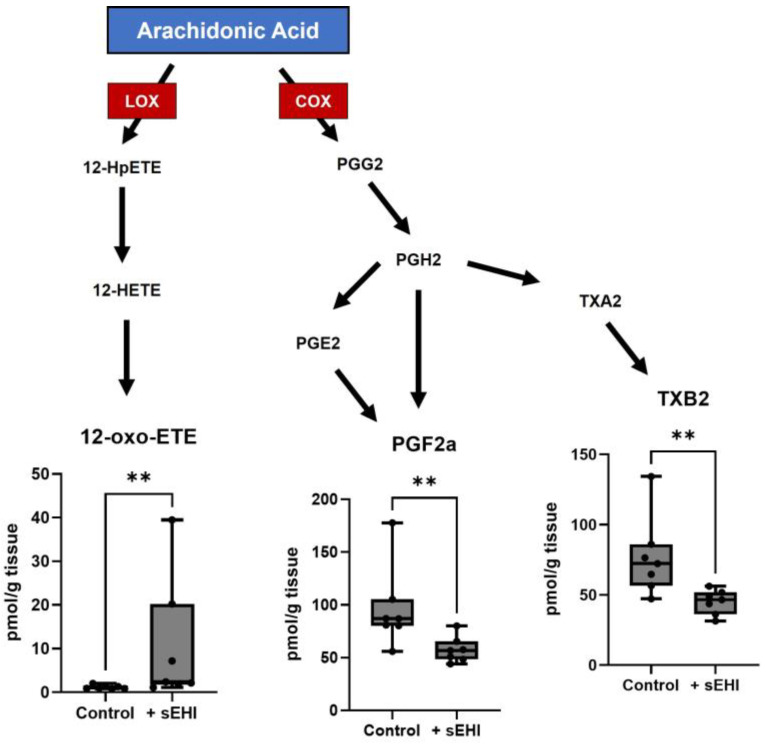
Oxylipins altered by sEHI in females. Boxplots of oxylipins that were significantly different in the brains of sEHI-treated and control female mice are shown in a diagram demonstrating the pathways producing the oxylipins from arachidonic acid. The box extends from the 25th to the 75th percentile, with the middle line indicating the median. The whiskers extend to the minimum and maximum values, with individual values indicated by black dots. For definitions of oxylipin abbreviations see Table S1. LOX: lipoxygenase enzyme; COX: cyclooxygenase enzyme; ** *p* < 0.01.

We analyzed the free oxylipin content of the brain in females with and without sEHI treatment in the same manner as in males. There were three oxylipins that were statistically different (*p* < 0.05) between sEHI-treated female mice and controls. All these oxylipins were products of arachidonic acid metabolism (Figure 5). 12-oxo-ETE, a product of LOX metabolism, was higher in the sEHI-treated mice than in controls. The two other oxylipins were both products of COX metabolism, PGF2a and TXB2, and were lower in the sEHI-treated mice than in controls.

### 3.3. Comparison of the Effect of sEHI on the Brain Oxylipin Profile in Males and Females

**Figure 6:**
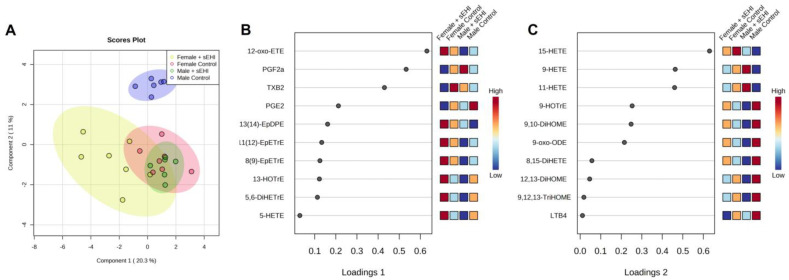
sPLS-DA of the oxylipin profiles of male and female control and sEHI-treated mice: (**A**) sPLS-DA plot of female sEHI-treated (color-coded yellow), female control (color-coded red), male sEHI-treated (color-coded green), and male control (color-coded blue) oxylipin profiles. (**B**) Loadings plot for the ten variables included in component one of the sPLS-DA. (**C**) Loadings plot for the ten variables included in component two of the sPLS-DA. For definitions of oxylipin abbreviations see Table S1.

With the aim to compare the impact of sEHI on the brain free oxylipin profile between males and females, we performed sPLS-DA to assess the separation between groups. The scores plot of the sPLS-DA demonstrated that both groups of females, with and without sEHI, clustered with the males receiving the sEHI, while control males without sEHI separated from the other three groups (Figure 6A). Loading plots, which show the variables selected by the sPLS-DA for a given component, can be seen in Figure 6B,C. The top three variables selected for component one matched the three oxylipins that were significantly altered by the sEHI in females, while all ten of the variables selected for component two were significantly altered by sEHI in males. 

**Figure 7:**
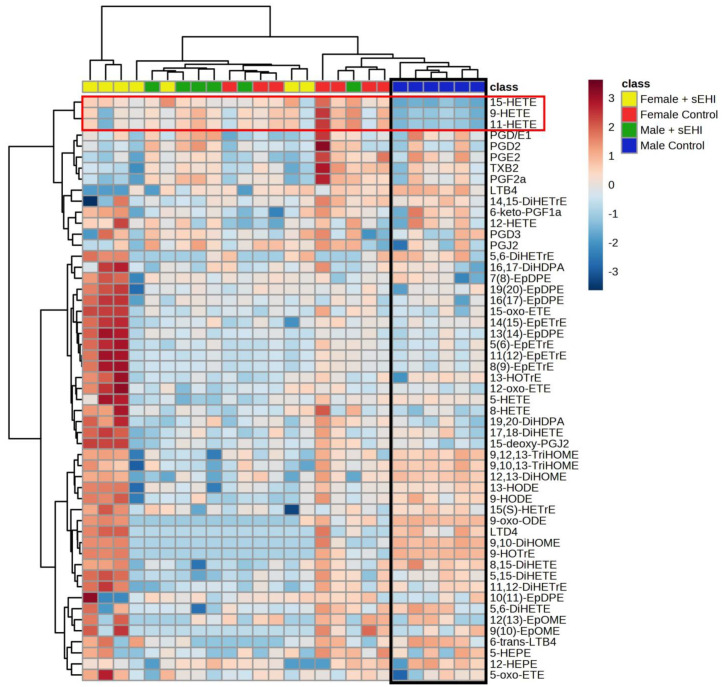
Hierarchical clustering heatmap of male and female control and sEHI-treated mice. The heatmap shows the relative concentrations of all measured oxylipins for the female sEHI-treated (color-coded yellow), female control (color-coded red), male sEHI-treated (color-coded green), and male control (color-coded blue) brain samples, with each column representing an individual sample. Throughout the heatmap, higher concentrations of the oxylipins are shown by shades of red and lower concentrations of oxylipins are shown by shades of blue. The black box highlights the clustering of control males together. The red box highlights three oxylipins that impact this clustering and are lower in control males. For definitions of oxylipin abbreviations see Table S1.

We chose to further analyze the relationship between response to sEHI and sex using a hierarchical clustering heatmap. In the heatmap, the male control mice cluster separately from all other groups, while the male sEHI-treated, as well as female control and sEHI-treated, mice were not distinctly separated by the clustering analysis (Figure 7). It can also be seen that three oxylipins (9-HETE, 11-HETE, and 15-HETE) defined the male control group cluster and were lower in concentration as compared to the other groups. These three oxylipins were also found to be significantly increased by sEHI in males by pairwise comparisons.

A summary of the concentrations of each oxylipin analyzed in males and females, with and without the inhibitor, can be found in Table S3.

### 3.4. sEHI Effects on Behavior and Cognition in Male and Female Mice

**Figure 8:**
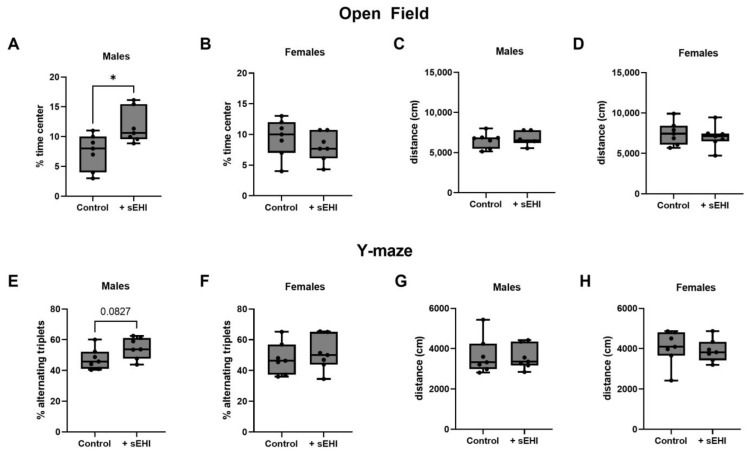
Behavioral and cognitive testing of sEHI-treated and control male and female mice. The percentage of time spent in the center of the open field test is shown for males (**A**) and females (**B**). The total distance traveled is shown for the open field test for males (**C**) and females (**D**). The percent alternating triplets during the Y-maze test is shown for males (**E**) and females (**F**). The total distance traveled is shown for the Y-maze for males (**G**) and females (**H**). The box extends from the 25th to the 75th percentile, with the middle line indicating the median. The whiskers extend to the minimum and maximum values, with individual values indicated by black dots. * *p* < 0.05.

We assessed behavior and cognitive function by the open field test and Y-maze test and only observed an effect of sEHI in male mice. Male mice treated with sEHI spent a greater percentage of time in the center of the open field test when than control male mice (Figure 8A). There was no difference between sEHI-treated and control female mice in the percent of time spent in the center of the open field test (Figure 8B). We saw no statistically significant differences between sEHI-treated mice and control mice in performance on the Y-maze test. There was a trend toward an increase in percent alternating triplets in sEHI-treated male mice compared to male controls, but it did not reach statistical significance (*p* = 0.0827) (Figure 8E). There was no difference between groups in females (Figure 8F). There was no difference in total distance traveled in both the open field test and the Y-maze test between the sEHI-treated and control groups in males or females (Figure 8C,D,G,H).

To further explore the relationship between the oxylipins and measures of behavior and cognitive function, we performed correlation analyses. As we only observed changes to behavior and cognitive function in males, we focused on the data from males, and oxylipins that were significantly altered by sEHI in males. We found a significant negative correlation between the percent time spent in the center on the open field test and 9-HOTrE (r = −0.6163, *p* = 0.0376), LTB4 (r = −0.599, *p* = 0.0433), 5,6-DiHETrE (r = −0.6532, *p* = 0.0252), and 14,15-DiHETrE (r = −0.6014, *p* = 0.0428) (Figure S2). We found that the percent alternating triplets in the Y-maze test negatively correlated with 13-HODE (r = −0.8182, *p* = 0.0019), 9,10-DiHOME (r = −0.6532, *p* = 0.0252), and 9,12,13-TriHOME (r = −0.6573, *p* = 0.0238) and positively correlated with 9-HETE (r = 0.5944, *p* = 0.0457) and 11-HETE (r = 0.5944, *p* = 0.0457) (Figure S3). 

## 4. Discussion

This study is the first comprehensive assessment of the brain oxylipin profile, and how it is impacted by inhibition of sEH in both sexes. Oxylipins are bioactive products of PUFA oxidation and have been demonstrated to play a role in neurological function, neuroinflammation, and neurodegenerative diseases, including dementias [8,11,12]. In addition, inhibition of sEH has been investigated as a potential therapeutic target for a wide range of neurological disorders, including dementias [13,21,22,26,30,31,34,35,36,37]. Therefore, the impact of sEHI on the comprehensive profile of oxylipins is an important area of study to further understand how this treatment might be beneficial in neurodegenerative diseases such as dementia, a major global killer of men and women. Importantly, we investigated sex differences, which are all too often overlooked and could provide insight into sex-specific treatments. By measuring the free oxylipin content of the brain with and without sEHI in males and females, we report an important novel finding; sEHI impacts the brain oxylipins of males differently, with a distinct pattern, and to a greater extent than in females. We discuss our findings in the context of prior work in the field, and implications for mechanisms, cognition, and therapeutics. 

### 4.1. Implications of Oxylipins Altered by sEHI in Males

We observed lower levels of the sEH products 5,6-DiHETrE, 14,15-DiHETrE, 9,10-DiHOME, and 12,13-DiHOME in sEHI-treated male mice compared to controls. These differences were to be expected and provide evidence that our inhibitor treatment was sufficient to inhibit sEH activity. Interestingly, 5,6-DiHETrE and 14,15-DiHETrE levels were negatively correlated with behavior indicative of reduced anxiety in the open field test, and 9,10-DiHOME levels were negatively correlated with memory function as measured by the Y-maze test. The DiHETrEs are the less biologically active metabolites of EpETrEs, and have been shown to have multiple protective effects in the brain, including anti-inflammatory effects, modulation of neuronal activity, regulation of blood flow, and improvement of cell survival for neurons and glial cells [41]. Further, serum levels of 12,13-DiHOME were previously shown to associate with white matter hyperintensities, an indicator of subcortical ischemic vascular damage [40]. Therefore, reductions in DiHETrE and DiHOME levels are a neuroprotective shift in the oxylipin profile.

We also observed differences in oxylipins not directly produced by sEH in male mice treated with the inhibitor. Most of these changes also appeared to be neuroprotective. 15-HETE is one of the oxylipins increased in the sEHI-treated group. 15-HETE is important for angiogenesis and recovery after MCAO and ischemia and has been shown to be present in lower levels in the brains of mice in an Alzheimer’s disease (AD) model [69,70,71,72]. Several oxylipins were lower in the sEHI-treated male brains compared to controls and have been previously found to be associated with brain injury or neurodegeneration: 5-HETE, LTB4, LTD4, and 9-HODE. 5-HETE was previously shown to be higher in the cerebrospinal fluid (CSF) of traumatic brain injury patients as compared to controls [73]. Brain levels of LTB4 have been associated with neuroinflammation and cognitive decline and were found to be increased in the CSF of AD patients [74,75]. In the current study, we also demonstrated that LTB4 levels were negatively correlated with behavior indicative of reduced anxiety in the open field test. LTD4 has been demonstrated to increase microglial activation, as well as facilitate amyloid β accumulation and cognitive impairment [76]. 9-HODE has previously been associated with white matter hyperintensities and reduced grey matter volume [10]. Thus, many of the differences seen in the brain oxylipin profiles of sEHI-treated and control male mice indicate a more neuroprotective oxylipin profile in the sEHI-treated mice.

### 4.2. Implications of Oxylipins Altered by sEHI in Females

In females, only three oxylipins were altered by sEHI treatment. None of these are a direct product of sEH; although, we did see a significant increase in the total EpETrE/DiHETrE ratio. The lack of significant changes in any of the individual products of sEH may be due to already low levels of sEH in females as reported previously [49,50]. The oxylipins found to be different between sEHI-treated and control mice were PGF2a, TXB2, and 12-oxo-ETE. PGF2a and TXB2 were decreased in mice receiving the inhibitor, while 12-oxo-ETE was increased. The existing literature suggests that reduced PGF2a levels may contribute to the neuropathology of dementias. PGF2a levels are reduced in the plasma and CSF of patients with AD and positively correlate with Mini-Mental State Examination scores [9,75]. Additionally, brain tissue from AD patients has a reduced capacity for the synthesis of PGF2a as compared to controls [77]. On the other hand, a reduction in TXB2 levels appears to be neuroprotective. TXB2 levels have been demonstrated to be higher in the brains of individuals with AD-like dementia, than in controls [78]. Additionally, patients with elevated levels of circulating TXB2 were found to have worse prognoses after stroke [79]. We were unable to find literature on the role of 12-oxo-ETE in the brain. Therefore, there were fewer changes in the free oxylipin composition of female sEHI-treated mice as compared to males, and the potential functional and mechanistic consequences are less clear than in males, partly due to the small number of oxylipins changed.

### 4.3. Sex Differences in the Response of the Brain Oxylipin Profile to sEHI

The sPLS-DA scores plot of the oxylipin content showed that male control and sEHI-treated groups were distinct, while the control and sEHI-treated female groups overlapped. Further, the hierarchical clustering heatmap demonstrated that the male sEHI-treated and control groups clustered separately, while sEHI-treated and control females did not. This would indicate that sEHI has a larger effect on the brain free oxylipin content of males as compared to females. Interestingly, none of the oxylipins altered by sEHI were common between females and males. The oxylipins that were altered in males were primarily downstream of LOX and CYP enzymatic activity, while the oxylipins altered in females were downstream of the LOX and COX. Others have demonstrated that sEH expression is higher in males, and that genetic deletion of sEH had a greater impact on reducing the consequences of cerebral ischemia in males than in females [49,50]. Thus, the sex differences in our study are supported by previous findings of the consequences of blocking sEH in the brain. In addition, the greater impact of inhibiting sEH in males could be explained by higher levels of the enzyme present at baseline in males. 

Our data suggest that treatment with sEHI shifts the brain oxylipin profile of males to be similar to that of females. In the sPLS-DA plot, the male control mice were distinct from the female mice, while the sEHI-treated male mice overlapped with both control and sEHI-treated females. Further, in the hierarchical clustering heatmap, only control males were distinctly clustered together. Within the heatmap, three of the oxylipins (9-HETE, 11-HETE, and 15-HETE) were visibly lower in the male control mice compared to all other groups. These three oxylipins were also found to be significantly increased by the sEHI in pairwise comparison. We previously demonstrated that these three oxylipins were higher in the brains of female mice than in male mice and were not affected by dietary sucrose content [65]. Although little is known about the functions of 9-HETE and 11-HETE, we saw a positive correlation between levels of 9-HETE and 11-HETE with memory as measured by the Y-maze test in males. Further, 15-HETE has been demonstrated to be neuroprotective [69,70,71,72]. Therefore, sEHI has a greater impact on the oxylipin profile of the brain in male mice, in part by shifting it to be more similar to that of female mice, thereby favoring less neurodegeneration.

### 4.4. Sex Differences in the Cognitive and Behavioral Outcomes with sEHI

The more robust response of males to the sEHI was also reflected in the results of the cognitive and behavioral tests of our study. We saw that, in males, sEHI-treated mice spent a greater amount of time in the center of the open field test, indicating a reduction in anxiety behavior [63], while no effect of sEHI was seen in females. In agreement with our findings, sEHI has previously been reported to increase the time spent in the center of the open field test in males [80]. We also saw a trend towards an increase in percent alternating triplets on the Y-maze test, suggesting improved working memory [64], in males; however, these changes were not observed in females. In agreement with our findings, an improvement in Y-maze test performance in a mouse model of AD when sEH was genetically deleted has been reported previously [39]. The sex of the mice studied was not specified; therefore, it is not possible to compare our sex differences findings to these results. Others have demonstrated that various sEHI compounds improve memory as measured by other cognitive function tests in the context of multiple disease models; however, these studies either only studied males, or combined males and females into one group [35,36,37,80,81,82,83,84,85]. Therefore, to our knowledge, our study is the first to describe sex differences in the effects of sEHI on murine behavior and memory.

### 4.5. Potential Mechanisms of sEHI Effects on Brain Oxylipins

Although four of the oxylipins altered by sEHI in males are direct products of sEH (5,6-DiHETrE, 14,15-DiHETrE, 9,10-DiHOME, and 12,13-DiHOME), the majority of the oxylipins that we found to differ between sEHI-treated mice and controls in males and females were not. This brings into question the mechanism behind the effect of sEHI on these other oxylipins. One possibility is that there are indirect effects of sEHI exerted through levels of its substrates and products. For example, EpETrEs have been shown to block the nuclear translocation of NFκB, which reduces expression of 5-LOX and COX-2 [13]. Many of the oxylipins that we found to be lower in the sEHI-treated male mice are downstream of 5-LOX. Additionally, PGF2a and TXB2, which were decreased in sEHI-treated females, are downstream of COX-2. Furthermore, knockout of sEH has been shown to increase the levels of 9-HETE, 11-HETE, and 15-HETE in plasma [86]. This supports our results and provides evidence that our findings are unlikely to be due to off-target effects; although, further research is needed. Therefore, the mechanism by which sEHI alters the level of oxylipins that are not direct substrates or products of sEH remains an important area for further study.

## 5. Conclusions

This study addressed a previously unexplored area of research—the response of the brain oxylipin profile to sEHI and the sex specificity in this response. We demonstrate that the oxylipin profile of the brain in males is impacted by sEHI to a greater extent than that of females. The changes induced by sEHI shift the brain oxylipin profile of males to be more similar to that of females and towards neuroprotection. Further, we show that this shift is associated with an improvement in behavior and memory in males. The dramatically different number of oxylipins impacted by sEHI between the sexes highlights the importance of research in both sexes. Further research addressing sex as a biologic variable may help to identify sex-specific treatment targets and strategies for neurodegenerative diseases such as dementia.

## Figures and Tables

**Table 1:** Bodyweight and serum analytes.

	Males		Females	
	Control	+ sEHI	Control	+ sEHI
Bodyweight (g)	36.19 ± 6.183	36.46 ± 5.384	29.20 ± 4.940	27.75 ± 2.559
Insulin (pg/mL)	230.5 ± 173.6	285.4 ± 142.6	208.2 ± 55.18	332.4 ± 170.4
Glucose (mg/dL)	416.1 ± 108.6	432.7 ± 128.5	282.9 ± 77.08	319.4 ± 117.9
Total Cholesterol (mg/dL)	154.9 ± 33.75	139.4 ± 14.35	89.74 ± 17.29	98.81 ± 13.56

Data shown are mean ± standard deviation.

## Data Availability

The data presented in this study are available in Table S3.

## Supplementary Materials

The following supporting information can be downloaded at https://www.mdpi.com/article/10.3390/nu15051214/s1, Table S1: List of measured oxylipins and internal standards with abbreviations. Table S2: Optimization parameters and parent and product ion monitoring pairs. Table S3: Summary of the concentrations of each oxylipin analyzed in males and females. Figure S1: Raw mass spectra for 15-HETE. Figure S2: Spearman correlation of open field percent time in center with significant oxylipins in males. Figure S3: Spearman correlation of Y-maze percent alternating triplets with significant oxylipins in males.
