# Supplementary material for: Sex-Specific Response of the Brain Free Oxylipin Profile to Soluble Epoxide Hydrolase Inhibition
Source: Nutrients. 2023 Feb 28;15(5):1214. doi: 10.3390/nu15051214 (PMC10005333; doi:10.3390/nu15051214)
Supplement: Supplementary file 1 [file nutrients-15-01214-s001.zip › nutrients-2228494-supplementary.pdf]

Supplementary Materials

Table S1. List of measured oxylipins and internal standards with abbreviations.

Compound name	Abbreviation
10(11)-epoxy-docosapentaenoic acid	10(11)-EpDPE
11(12)-epoxy-eicosatetraenoic acid	11(12)-EpETE
11(12)-epoxy-eicosatrienoic acid	11(12)-EpETrE
11,12-dihydroxy-eicosatetraenoic acid	11,12-DiHETE
11,12-dihydroxy-eicosatrienoic acid	11,12-DiHETrE
11-hydroxy-eicosatetraenoic acid	11-HETE
12(13)-epoxy-octadecamonoenoic acid	12(13)EpOME
12,13-dihydroxy-octadecamonoenoic acid	12,13-DiHOME
12-hydroxy-eicosapentaenoic acid	12-HEPE
12-hydroxy-eicosatetraenoic acid	12-HETE
12-oxo-eicosatetraenoic acid	12-oxo-ETE
13(14)-epoxy-docosapentaenoic acid	13(14)-EpDPE
13-hydroxy-octadecadienoic acid	13-HODE
13-hydroxy-octadecatrienoic acid	13-HOTrE
13-oxo-octadecadienoic acid	13-oxo-ODE
14(15)-epoxy-eicosatetraenoic acid	14(15)-EpETE
14(15)-epoxy-eicosatrienoic acid	14(15)-EpETrE
14,15-dihydroxy-eicosatetraenoic acid	14,15-DiHETE
14,15-dihydroxy-eicosatrienoic acid	14,15-DiHETrE
15(S)-hydroxy-eicosatrienoic acid	15(S)-HETrE
15-deoxy-Prostaglandin J2	15-deoxy-PGJ2
15-hydroxy-eicosapentaenoic acid	15-HEPE
15-hydroxy-eicosatetraenoic acid	15-HETE
15-oxo-eicosatetraenoic acid	15-oxo-ETE
16(17)-epoxy-docosapentaenoic acid	16(17)-EpDPE
16,17-dihydroxy-docosapentaenoic acid	16,17 DiHDPA
17- hydroxy-docosahexaenoic acid	17-HDoHE
17(18)-epoxy-eicosatetraenoic acid	17(18)-EpETE
17,18-dihydroxy-eicosatetraenoic acid	17,18-DiHETE
19(20)-epoxy-docosapentaenoic acid	19(20)-EpDPE
19,20-dihydroxy-docosapentaenoic acid	19,20-DiHDPA
20-COOH- Leukotriene B4	20-COOH-LTB4
20-hydroxy-eicosatetraenoic acid	20-HETE

20-OH-Leukotriene B4	20-OH-LTB4
5(6)-epoxy-eicosatrienoic acid	5(6)-EpETrE
5,15-dihydroxy-eicosatetraenoic acid	5,15-DiHETE
5,6-dihydroxy-eicosatetraenoic acid	5,6-DiHETE
5,6-dihydroxy-eicosatrienoic acid	5,6-DiHETrE
5-hydroxy-eicosapentaenoic acid	5-HEPE
5-hydroxy-eicosatetraenoic acid	5-HETE
5-oxo-eicosatetraenoic acid	5-oxo-ETE
6-keto-prostaglandin F1-α	6-keto-PGF1a
6-trans-Leukotriene B4	6-trans-LTB4
7(8)-epoxy-docosapentaenoic acid	7(8)-EpDPE
8(9)-epoxy-eicosatetraenoic acid	8(9)-EpETE
8(9)-epoxy-eicosatrienoic acid	8(9)-EpETrE
8,15-dihydroxy-eicosatetraenoic acid	8,15-DiHETE
8,9-dihydroxy-eicosatetraenoic acid	8,9-DiHETE
8,9-dihydroxy-eicosatrienoic acid	8,9-DiHETrE
8-hydroxy-eicosapentaenoic acid	8-HEPE
8-hydroxy-eicosatetraenoic acid	8-HETE
9(10)-epoxy-octadecamonoenoic acid	9(10)-EpOME
9,10,13-trihydroxy-octadecamonoenoic acid	9,10,13-TriHOME
9,10-dihydroxy-octadecamonoenoic acid	9,10-DiHOME
9,12,13-trihydroxy-octadecamonoenoic acid	9,12,13-TriHOME
9-hydroxy-eicosatetraenoic acid	9-HETE
9-hydroxy-octadecadienoic acid	9-HODE
9-hydroxy-octadecatrienoic acid	9-HOTrE
9-oxo-octadecadienoic acid	9-oxo-ODE
d11-11(12)-epoxy-eicosatrienoic acid	d-11-11(12)EpETrE
d11-14,15-dihydroxy-eicosatrienoic acid	d11-14,15-DiHETrE
d4-6-keto-prostaglandin F1-α	d4-6-keto-PGF1a
d4-9-hydroxy-octadecadienoic acid	d4-9HODE
d4-Leukotriene B4	d4-LTB4
d4-Prostaglandin E2	d4-PGE2
d-4-Thromboxane B2	d4-TXB2
d6-20-hydroxy-eicosatetraenoic acid	d6-20-HETE
d8-5-hydroxy-eicosatetraenoic acid	d8-5-HETE
Leukotriene B3	LTB3
Leukotriene B4	LTB4
Leukotriene C4	LTC4
Leukotriene D4	LTD4

Leukotriene E4	LTE4
Lipoxin A4	LXA4
Prostaglandin B2	PGB2
Prostaglandin D1+ E1 combined	PGD/E1
Prostaglandin D2	PGD2
Prostaglandin D3	PGD3
Prostaglandin E2	PGE2
Prostaglandin E3	PGE3
Prostaglandin F2-α	PGF2a
Prostaglandin J2	PGJ2
Resolvin E1	Resolvin E1
Thromboxane B2	TXB2

Table S2. Optimization parameters and parent and product ion monitoring pairs.

Compound	Prec Ion (m/z)	Prod Ion (m/z)	Frag (V)	CE (V)	Cell Acc (V)	Ret Time (min)	Internal Standard
Measured Oxylipins							
20-COOH-LTB4	365.2	347.2	110	10	4	3.78	d4-6-keto-PGF1a
Resolvin E1	349.3	195	90	10	4	3.902	d4-6-keto-PGF1a
6-keto-PGF1a	369.3	163.2	80	7	4	3.988	d4-6-keto-PGF1a
20-OH-LTB4	351.2	195.2	130	12	4	4.08	d4-6-keto-PGF1a
PGE3	349.3	269.2	80	5	4	4.986	d4-PGE2
PGD3	349.3	269.2	80	5	4	5.25	d4-PGE2
TXB2	369.2	169.1	80	5	4	5.3	d4-TXB2
9,12,13-TriHOME	329.2	211.1	120	8	4	5.479	d4-PGE2
9,10,13-TriHOME	329.2	171.1	120	8	4	5.581	d4-PGE2
PGF2a	353.2	309.2	80	12	4	5.601	d4-PGE2
PGD/E1	353.3	317.2	80	5	4	5.8	d4-PGE2
PGE2	351.2	271.3	80	10	4	5.837	d4-PGE2
PGD2	351.2	271.3	80	10	4	6.1	d4-PGE2
LTD4	495.3	177.1	110	12	4	6.375	d4-LTB4
LXA4	351.2	115.2	90	10	4	6.691	d4-LTB4
LTE4	438.2	333.1	80	5	4	7.687	d4-LTB4
PGJ2	333.3	189.2	100	12	4	7.956	d4-PGE2
LTC4	624.3	272.1	110	12	4	8	d4-LTB4
PGB2	333.3	175.1	80	10	4	8.042	d4-PGE2
LTB4	335.2	195.1	80	7	4	8.6	d4-LTB4
8,15-DiHETE	335.2	235.2	50	7	4	8.606	d11-14,15-DiHETrE

6-trans-LTB4	335.2	195.1	80	7	4	8.865	d4-LTB4
5,15-DiHETE	335.2	173.2	90	7	4	8.875	d11-14,15-DiHETrE
17,18-DiHETE	335.3	247.2	80	7	4	9	d11-14,15-DiHETrE
14,15-DiHETE	335.3	207.2	100	10	4	9.33	d11-14,15-DiHETrE
11,12-DiHETE	335.5	167	100	10	4	9.46	d11-14,15-DiHETrE
12,13-DiHOME	313.2	183.2	110	12	4	9.6	d11-14,15-DiHETrE
8,9-DiHETE	335.5	126.9	100	12	4	9.699	d11-14,15-DiHETrE
9,10-DiHOME	313.2	201.2	120	12	4	9.871	d11-14,15-DiHETrE
5,6-DiHETE	335.2	115.2	90	12	4	9.899	d11-14,15-DiHETrE
14,15-DiHETrE	337.2	207.1	100	5	4	10.2	d11-14,15-DiHETrE
19,20-DiHDPA	361.5	273.1	100	8	4	10.2	d11-14,15-DiHETrE
LTB3	337.2	195.2	110	7	4	10.31	d4-LTB4
16,17 DiHDPA	361.5	233.1	100	8	4	10.5	d11-14,15-DiHETrE
11,12-DiHETrE	337.2	167.1	120	12	4	10.62	d11-14,15-DiHETrE
9-HOTrE	293.2	171.2	120	10	4	10.765	d4-9HODE
13-HOTrE	293.2	195.1	100	10	4	10.95	d4-9HODE
8,9-DiHETrE	337.2	127.1	70	12	4	10.963	d11-14,15-DiHETrE
15-deoxy-PGJ2	315.2	271.2	120	5	4	11.2	d4-PGE2
15-HEPE	317.2	219.2	110	5	4	11.28	d6-20-HETE
20-HETE	319.2	275.1	110	7	4	11.3	d6-20-HETE
5,6-DiHETrE	337.2	145.1	90	12	4	11.43	d11-14,15-DiHETrE
8-HEPE	317.2	155.2	100	7	4	11.448	d6-20-HETE
12-HEPE	317.2	179.2	110	7	4	11.53	d6-20-HETE
5-HEPE	317.2	115.1	80	5	4	11.78	d6-20-HETE
13-HODE	295.2	195.2	100	7	4	11.89	d4-9HODE
9-HODE	295.2	171.1	110	12	4	11.951	d4-9HODE
15-HETE	319.2	219.2	120	5	4	12.171	d8-5-HETE
17(18)-EpETE	317.2	215.2	90	10	4	12.27	d-11-11(12)EpEtrE
13-oxo-ODE	293.2	195.1	120	10	4	12.336	d4-9HODE
11-HETE	319.2	167.2	100	10	4	12.4	d8-5-HETE
9-HETE	319.2	167.2	100	10	4	12.414	d8-5-HETE
15-oxo-ETE	317.2	113.1	110	7	6	12.5	d8-5-HETE
14(15)-EpETE	317.2	207.2	90	5	4	12.575	d-11-11(12)EpEtrE
9-oxo-ODE	293.2	185.1	120	10	4	12.579	d4-9HODE
12-HETE	319.2	179.2	100	10	4	12.62	d8-5-HETE
8-HETE	319.2	155.2	90	5	4	12.624	d8-5-HETE
11(12)-EpETE	317.2	167.2	100	10	4	12.65	d-11-11(12)EpEtrE
8(9)-EpETE	317.2	127.2	100	10	4	12.752	d-11-11(12)EpEtrE
15(S)-HETrE	321.2	221.2	80	10	4	12.8	d8-5-HETE

12-oxo-ETE	317.2	153.1	70	7	4	12.885	d8-5-HETE
5-HETE	319.2	115.1	80	5	4	12.96	d8-5-HETE
17-HDoHE	343.2	281.2	50	7	4	13.22	d4-9HODE
19(20)-EpDPE	343.2	241.2	120	5	4	13.23	d-11-11(12)EpEtrE
12(13)EpOME	295.3	195.2	100	7	4	13.373	d-11-11(12)EpEtrE
14(15)-EpETrE	319.2	219.3	120	5	4	13.4	d-11-11(12)EpEtrE
9(10)-EpOME	295.3	171.2	100	10	4	13.49	d-11-11(12)EpEtrE
16(17)-EpDPE	343.2	233.2	70	5	4	13.5	d-11-11(12)EpEtrE
13(14)-EpDPE	343.2	193.2	70	5	4	13.53	d-11-11(12)EpEtrE
10(11)-EpDPE	343.2	153.2	90	5	4	13.62	d-11-11(12)EpEtrE
5-oxo-ETE	317.2	273.2	120	5	4	13.648	d8-5-HETE
8(9)-EpETrE	319.2	167.2	50	7	4	13.755	d-11-11(12)EpEtrE
11(12)-EpETrE	319.2	167.2	100	10	4	13.76	d-11-11(12)EpEtrE
7(8)-EpDPE	343.2	113.1	50	5	4	13.761	d-11-11(12)EpEtrE
5(6)-EpETrE	319.2	191.1	120	5	4	14.04	d-11-11(12)EpEtrE
Internal Standards							
d4-6-keto-PGF1a	373.3	167.1	110	25	4	3.937	NA
d4-TXB2	373.3	173.2	100	12	4	5.5	NA
d4-PGE2	355.2	275.3	100	10	4	5.817	NA
d4-LTB4	339.2	197.2	80	10	4	9.129	NA
d11-14,15-DiHETrE	348.2	207.1	110	12	6	10.125	NA
d6-20-HETE	325.2	281.2	70	12	4	11.279	NA
d4-9HODE	299.2	172.3	80	10	4	11.9	NA
d8-5-HETE	327.2	116.1	70	7	4	12.881	NA
d-11-11(12)EpETrE	330.2	167.2	130	7	4	13.678	NA

Table S3. Summary of the concentrations of each oxylipin analyzed in males and females.

Oxylipin	Male Control	Male + sEHI	Female Control	Female + sEHI
6-keto-PGF1a	70.62 (23.34)	54.87 (9.95)	51.99 (18.77)	77.34 (32.14)
TXB2	61.60 (12.35)	63.89 (4.05)	72.37 (20.59)	46.62 (10.41)*
PGD3	0.56 (0.30)	0.68 (0.11)	0.47 (0.13)	0.61 (0.32)
9,12,13-TriHOME	1.41 (0.46)	0.44 (0.38)#	0.70 (0.64)	0.29 (2.80)
9,10,13-TriHOME	0.94 (0.15)	0.26 (0.45)#	0.46 (0.24)	0.24 (0.99)
PGF2a	74.05 (17.84)	94.73 (15.17)	86.94 (15.60)	56.80 (11.96)*
PGE2	47.61 (20.92)	47.93 (3.53)	46.97 (28.66)	34.15 (9.03)
PGD/E1	1.90 (0.50)	2.17 (0.36)	1.62 (0.41)	1.59 (0.27)
PGD2[1]	253.53 (85.03)	355.64 (40.18)	266.07 (65.44)	263.86 (34.89)
LTD4	0.085 (0.18)	0.0014 (0.0000)	0.0014 (0.0000)	0.0014 (2.84)

PGJ2	1.56 (0.73)	2.17 (0.55)#	2.24 (0.89)	1.64 (0.45)
8,15-DiHETE	58.71 (11.71)	17.97 (13.35)#	33.71 (32.39)	42.62 (70.32)
6-trans-LTB4	0.39 (0.46)	0.023 (0.049)*	0.0075 (0.27)	0.060 (0.57)
5,6-DiHETE	8.91 (4.87)	4.48 (1.48)	6.65 (4.99)	4.44 (3.49)
5,15-DiHETE	2.31 (0.49)	1.45 (0.36)*	2.39 (1.14)	2.15 (4.45)
17,18-DiHETE	4.56 (2.76)	1.93 (1.46)	3.90 (3.72)	3.59 (32.30)
LTB4	0.59 (0.25)	0.16 (0.22)*	0.26 (0.045)	0.21 (0.31)
12,13-DiHOME	0.76 (0.56)	0.025 (0.13)#	0.25 (0.33)	0.084 (2.06)
9,10-DiHOME	0.38 (0.14)	0.0061 (0.0000)#	0.0061 (0.037)	0.0061 (1.35)
19,20-DiHDPA	0.91 (0.31)	1.14 (0.47)	1.28 (0.42)	1.33 (2.12)
14,15-DiHETrE	1.13 (0.16)	0.77 (0.30)*	1.28 (0.67)	0.62 (0.38)
16,17-DiHDPA	0.19 (0.10)	0.11 (0.053)	0.20 (0.11)	0.18 (0.49)
11,12-DiHETrE	0.43 (0.17)	0.23 (0.093)	0.34 (0.26)	0.22 (1.12)
9-HOTrE	1.70 (0.40)	0.0066 (0.0000)#	0.0066 (0.26)	0.0066 (11.99)
13-HOTrE	1.45 (0.27)	0.49 (0.11)	0.73 (0.81)	1.06 (9.53)
15-deoxy-PGJ2	0.57 (0.23)	0.69 (0.31)	0.74 (0.51)	0.60 (5.52)
5,6-DiHETrE	0.079 (0.086)	0.0043 (0.0000)*	0.0043 (0.049)	0.13 (0.29)
12-HEPE	0.61 (0.45)	0.37 (0.30)	0.40 (0.17)	0.20 (0.21)
5-HEPE	0.17 (0.24)	0.029 (0.074)	0.059 (0.56)	0.076 (0.30)
13-HODE	7.37 (1.99)	4.20 (2.06)#	5.17 (1.90)	4.97 (32.96)
9-HODE	4.78 (0.27)	2.13 (0.20)*	3.10 (1.72)	3.03 (10.94)
15-HETE	104.50 (8.10)	244.39 (41.76)#	260.90 (35.82)	283.16 (112.06)
9-HETE	91.16 (10.25)	181.73 (43.42)#	198.92 (75.76)	145.53 (40.86)
11-HETE	91.28 (10.37)	181.76 (43.33)#	198.87 (76.01)	141.96 (40.56)
15-oxo-ETE	5.98 (3.15)	7.74 (3.01)	7.12 (3.96)	6.70 (54.80)
9-oxo-ODE	0.98 (0.21)	0.00069 (0.00000)#	0.0035 (0.16)	0.18 (6.41)
8-HETE	10.35 (3.73)	13.96 (1.75)	11.33 (2.05)	18.54 (12.72)
12-HETE	100.50 (60.29)	109.33 (55.14)	52.05 (26.65)	82.75 (26.49)
15(S)-HETrE	2.81 (0.80)	1.99 (0.97)	1.29 (0.79)	3.02 (5.69)
12-oxo-ETE	1.17 (0.37)	0.87 (0.47)	0.99 (0.52)	2.38 (11.77)*
5-HETE	14.46 (1.50)	7.05 (1.94)#	12.52 (3.05)	12.69 (31.60)
19(20)-EpDPE	4.86 (1.83)	3.84 (0.75)	6.22 (4.13)	5.96 (205.81)
12(13)-EpOME	0.47 (2.84)	0.048 (0.14)	2.64 (2.75)	0.048 (10.92)
14(15)-EpETrE	23.05 (6.69)	19.78 (4.99)	25.64 (7.72)	21.57 (147.11)
16(17)-EpDPE	2.60 (1.55)	2.97 (0.55)	1.97 (2.21)	3.30 (162.40)
9(10)-EpOME	0.11 (0.16)	0.033 (0.0000)	0.16 (1.15)	0.033 (2.45)
13(14)-EpDPE	2.48 (0.87)	3.16 (1.45)	3.36 (1.18)	3.97 (107.54)
10(11)-EpDPE	1.23 (1.49)	2.90 (1.47)	2.91 (1.76)	2.76 (2.96)
5-oxo-ETE	4.11 (2.87)	2.81 (2.05)	4.12 (2.05)	10.79 (8.80)

7(8)-EpDPE	2.68 (4.34)	2.37 (2.31)	2.70 (1.75)	2.99 (108.05)
11(12)-EpETrE	19.13 (8.21)	17.28 (4.19)	21.53 (7.59)	17.84 (160.69)
8(9)-EpETrE	21.37 (8.37)	19.93 (3.26)	23.66 (7.61)	19.84 (160.25)
5(6)-EpETrE	21.13 (11.17)	22.96 (8.70)	24.10 (10.13)	21.24 (169.77)

Median (interquartile range) all units: pmol/g tissue.

* p<0.05 compared to control mice of the same sex; # FDR adjusted p<0.05 compared to control mice of the same sex; [1] PGD2 signal was above the standard curve for many samples.

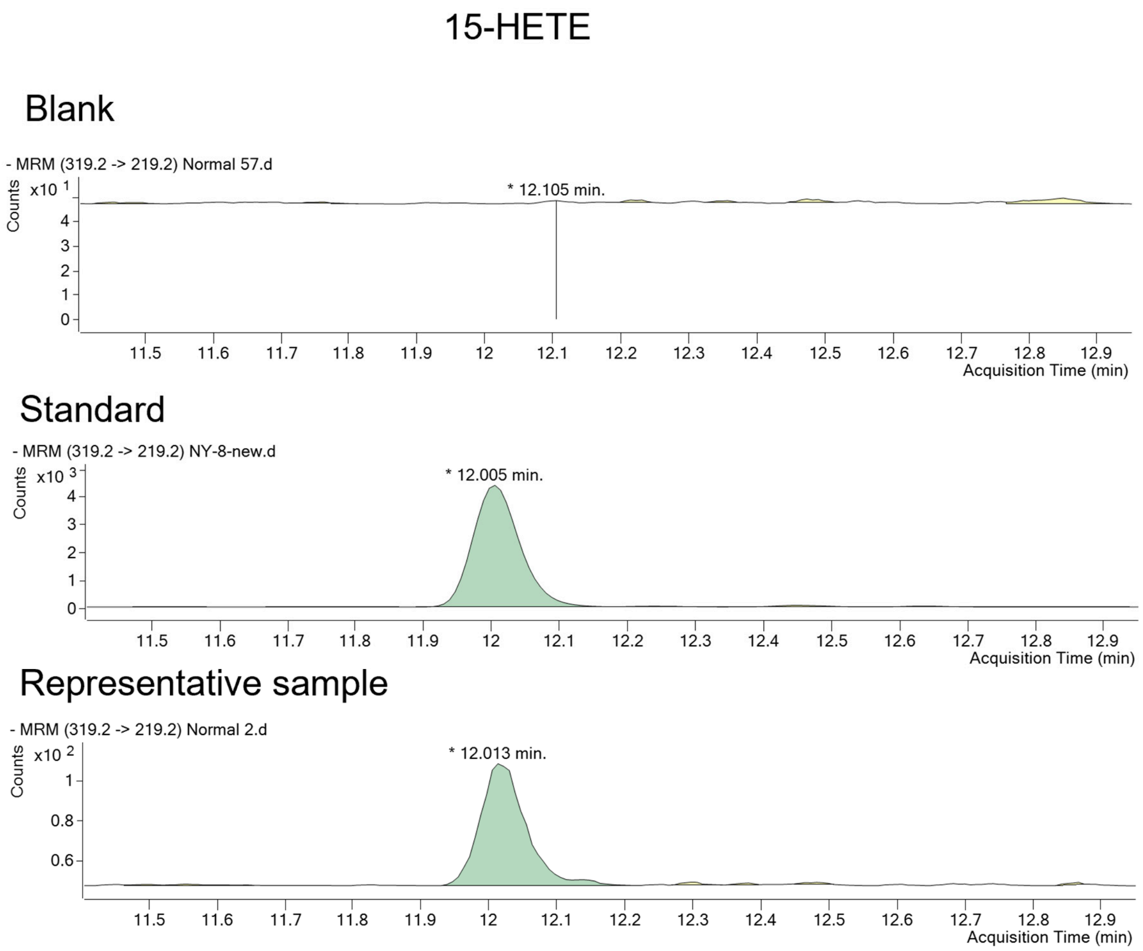

Figure S1. Raw mass spectra for 15-HETE. Raw mass spectra are provided for blank, standard, and a representative sample for 15-HETE.

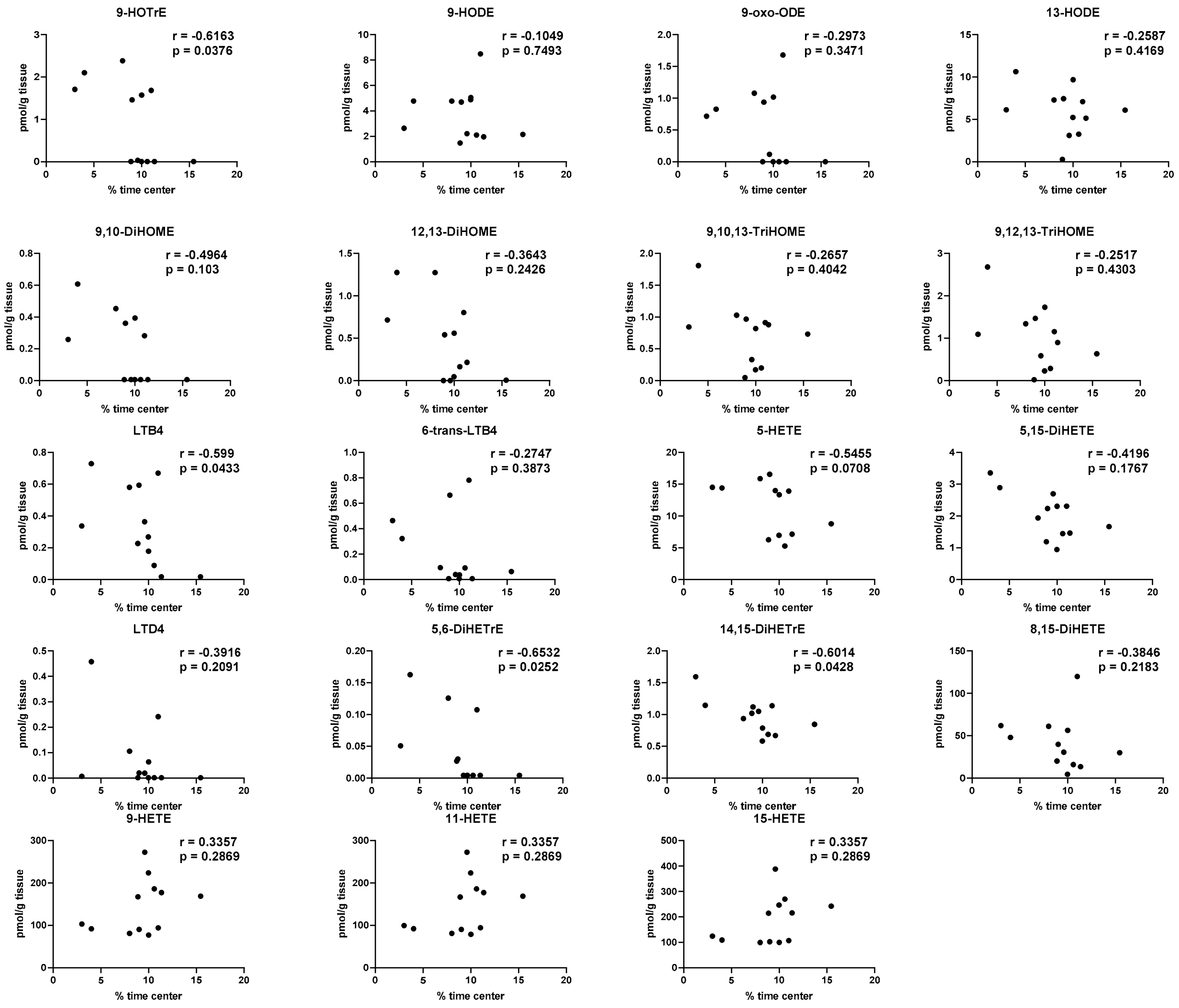

Figure S2. Spearman correlation of open field percent time in center with significant oxylipins in males. Scatter plots of male data of open field percent time in center and oxylipin concentrations for all oxylipins significantly changed by sEHI in males. The Spearman r and two-tailed p value are indicated on each scatter plot.

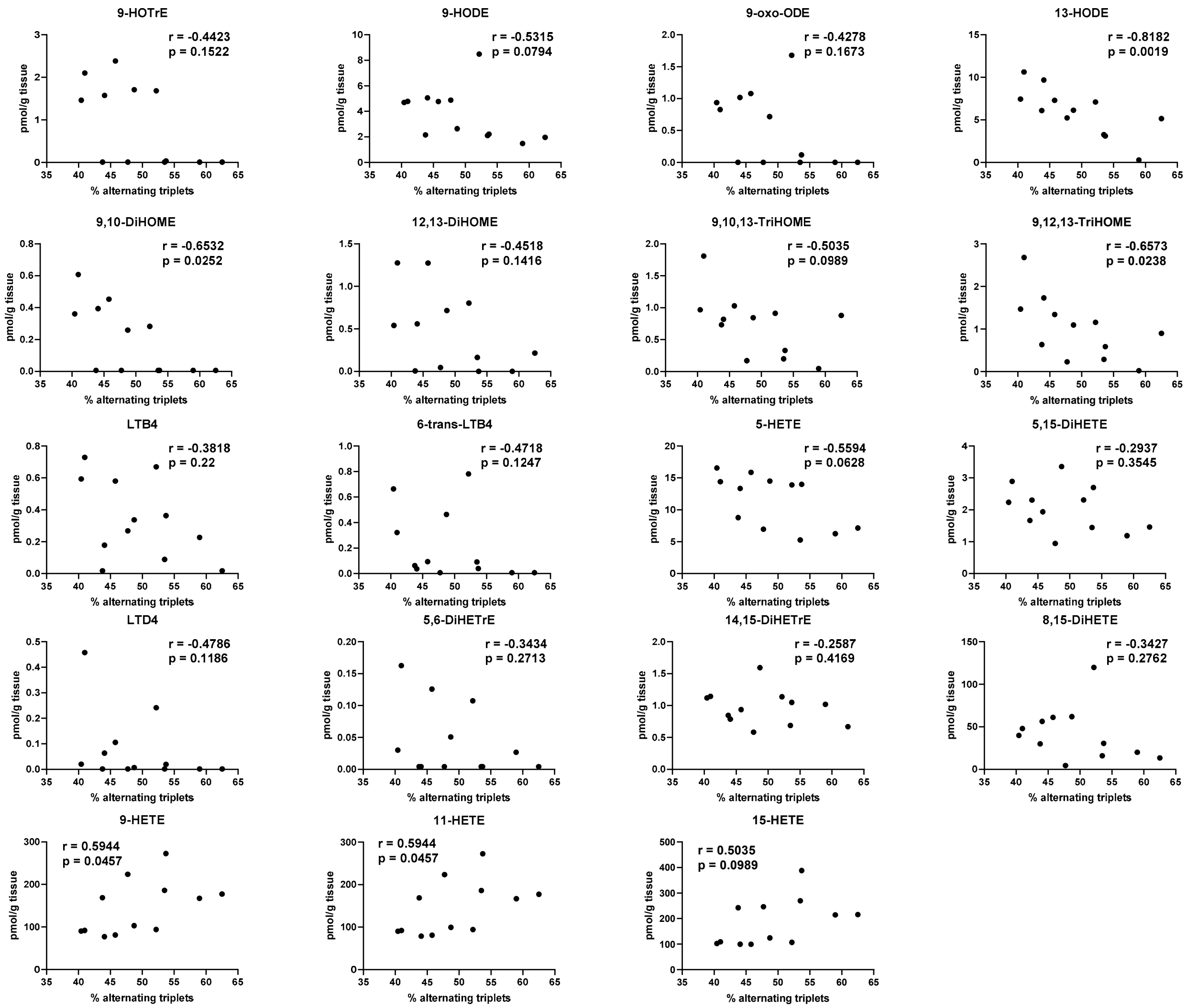

Figure S3. Spearman correlation of Y-maze percent alternating triplets with significant oxylipins in males. Scatter plots of male data of Y-maze percent alternating triplets and oxylipin concentrations for all oxylipins significantly changed by sEHI in males. The Spearman r and two-tailed p value are indicated on each scatter plot.
